# Routine Chest X-Rays in Critical Bronchiolitis Do Not Improve Outcomes

**DOI:** 10.3390/jcm14217810

**Published:** 2025-11-03

**Authors:** Trisha Sunderajan, Da-Eun (Shira) Choi, Caroline LaFerla, Robert D. Guglielmo, Harsha K. Chandnani, Michael C. Mount, Harmanpreet S. Chawla, Michael E. Giang

**Affiliations:** 1 Division of Pediatric Critical Care, Department of Pediatrics, Blank Children’s Hospital, Des Moines, IA 50309, USA; 2 Department of Pediatrics, Loma Linda University School of Medicine, Loma Linda University Children’s Hospital, Loma Linda, CA 92354, USA; daeunchoi@llu.edu (D.-E.C.); claferla@llu.edu (C.L.); 3 Division of Pediatric Critical Care, Department of Pediatrics, Loma Linda University School of Medicine, Loma Linda University Children’s Hospital, Loma Linda, CA 92354, USA; rguglielmo@llu.edu (R.D.G.); hchandnani@llu.edu (H.K.C.); hchawla@llu.edu (H.S.C.); mgiang@llu.edu (M.E.G.); 4 Division of Pediatric Critical Care, Department of Pediatrics, Children’s Hospital of Atlanta, Atlanta, GA 30329, USA; mcmount@emory.edu

**Keywords:** critical bronchiolitis, non-invasive ventilation, CXR, critical bronchiolitis score

## Abstract

**Background:** Routine chest X-rays (CXR) are not recommended by the American Academy of Pediatrics in bronchiolitis, yet remain a mainstay in diagnostics. We aimed to understand the impact of obtaining CXRs in patients with critical bronchiolitis, assessing intensive care unit length of stay (ICU-LOS) and intensive care unit level of respiratory support (ICU-LRS). **Methods/Design:** This single-center retrospective cohort study assessed children less than three years of age admitted to the PICU, pediatric step-down ICU, and pediatric cardiac ICU. Two groups were used for analysis: patients with CXR and no-CXR. The primary outcome was the difference in ICU-LOS and ICU-LRS between the groups. The critical bronchiolitis score (CBS) was used to calculate a predicted ICU-LOS and ICU-LRS. The secondary outcome was the difference between actual and predicted ICU-LOS and ICU-LRS, comparing the groups. **Results:** Of the 107 patients included, 65 patients (61%) received a CXR. Patients who received a CXR had significantly longer ICU-LOS (*p* = 0.01) and ICU-LRS (*p* = 0.02), despite no difference in predicted illness severity (ICU-LOS, *p* = 0.4; ICU-LRS, *p* = 0.3). The difference between actual and predicted ICU-LOS was greater in the no-CXR group (–1.4 days) compared to the CXR group (–0.8 days; *p* = 0.04). A similar trend was observed in ICU-LRS (–0.1 vs. –0.6 days; *p* = 0.1), though not statistically significant. **Conclusions:** Routine CXRs are common in critically ill bronchiolitis patients and may be associated with longer ICU-LOS and ICU-LRS, despite similar illness severity.

## 1. Introduction

Bronchiolitis is the leading cause of hospital admissions in children, yet effective therapies and diagnostics beyond supportive care remain controversial [1]. Despite the American Academy of Pediatrics (AAP) and other international guidelines discouraging routine chest X-rays (CXRs) [1,2,3], they are still used in 83–98% of PICU cases, often without altering clinical management [4].

Routine CXRs in bronchiolitis reveal non-specific findings, such as atelectasis or infiltrates, which are frequently misinterpreted as bacterial pneumonia. Excess imaging most directly leads to unnecessary radiation exposure, but can also lead to radiographic misinterpretations, such as suggesting bacterial pneumonia, and thus, otherwise avoidable and potentially unnecessary treatments, such as antibiotics [5,6]. This also contributes to high healthcare costs [5]. The economic burden of bronchiolitis is significant, costing the U.S. healthcare system $1.73 billion annually [7]. Evidence suggests that reducing unnecessary radiography can lower costs without compromising the diagnostic accuracy of bacterial pneumonia [7].

While the AAP’s “less is more” approach effectively manages non-severe bronchiolitis, it does acknowledge disease heterogeneity, stating that “initial radiography should be reserved for cases in which respiratory effort is severe enough to warrant ICU admission” [8]. However, current evidence-based protocols primarily address non-critically ill children [9,10] or children in the ED [11], leading to variability in imaging practices in those admitted to the ICU. Even when evaluated for findings such as fevers or hypoxemia, the yield of routine imaging in bronchiolitis is low [12].

This study aimed to evaluate the impact of routine CXRs in critical bronchiolitis, specifically focusing on ICU length of stay (ICU-LOS) and the level of respiratory support (ICU-LRS). Critical bronchiolitis was defined as patients diagnosed with viral bronchiolitis requiring ICU-LRS, including high-flow nasal cannula therapy (HFNC), non-invasive mechanical ventilation (NIMV), Continuous Positive Airway Pressure (CPAP), Bilevel Positive Airway Pressure (BIPAP), or invasive mechanical ventilation (IMV); as these therapies, in most institutions, would warrant an ICU admission.

## 2. Materials and Methods

### 2.1. Study Overview

We performed a single-center retrospective study at Loma Linda University Children’s Hospital. We included children aged 0–36 months admitted to the PICU, step-down ICU (SDICU), or pediatric cardiac ICU (PCTICU) with a clinical diagnosis of critical bronchiolitis requiring an ICU level of Respiratory Support (ICU-LRS). ICU-LRS was defined as requiring high-flow nasal cannula therapy (HFNC), non-invasive mechanical ventilation (NIMV), Continuous Positive Airway Pressure (CPAP), Bilevel Positive Airway Pressure (BIPAP), or invasive mechanical ventilation (IMV). We utilized the Virtual Pediatric Systems (VPS) ICU database as well as the Epic electronic medical record to identify patients via bronchiolitis diagnosis code.

We utilized the Critical Bronchiolitis Score (CBS), a validated tool that predicts ICU-LRS and ICU-LOS, to control for illness severity. The CBS is a score derived from a retrospective database study spanning 128 North American PICUs, utilizing 14,478 children admitted to PICUs with a primary diagnosis of bronchiolitis and with ICU-level of respiratory support. It utilizes 12 patient-level variables to determine ICU-level respiratory support and also length of stay [13]. We extended the age cut-off to 36 months to reflect real-world practice, as physicians often treated children just over 24 months as bronchiolitis, and excluding them risked introducing selection bias by omitting clinically similar cases.

The study period spanned from 1 January 2022 to 31 December 2022. Children with a history of cardiac surgery within seven days, readmission to PICU during the same hospitalization, tracheostomy dependence, and age greater than 3 years were excluded. As our aim was to determine the impact of acute viral bronchiolitis, patients who were exposed to cardiac surgery were excluded in order to remove the confounding variable of cardiac dysfunction. Similarly, if patients had a readmission to the PICU during the same hospitalization, we would not expect this to be typical of the course of acute viral bronchiolitis, and therefore these patients were excluded. The study was approved by the Institutional Review Board at Loma Linda University Children’s Hospital.

### 2.2. Outcome Measure

The primary outcome measures were ICU-LOS and ICU-LRS. Patients were divided into two groups for analysis: those who received CXRs and those who did not. CXRs were counted as received if they occurred in the ER prior to or during the ICU admission. Actual ICU-LOS and ICU-LRS were measured for patients admitted to the PICU, SDICU, and PCTICU.

The predicted ICU-LOS and ICU-LRS were calculated for both the CXR and no-CXR groups using the Critical Bronchiolitis Score (CBS) [13]. The difference between actual and predicted values for ICU-LOS and ICU-LRS was then analyzed to compare the effect on outcomes between the two groups.

### 2.3. Data Collection

Variables collected from each patient included demographic data, past medical history, past surgical history, home-oxygen use, day-of-illness on presentation, month and year of presentation, presence of viral infection, ICU-LOS, hospital LOS, type and duration of respiratory support, CXR status, and use of antibiotics, bronchodilators, steroids, or nebulized hypertonic saline. Data was also collected for additional imaging modalities, including lung ultrasound, chest computed tomography (CT), and echocardiography, with associated relevant findings.

To evaluate the CXR findings, a scoring system was used. Each CXR was assessed for actionable findings (defined as the presence of consolidation, pneumothorax, or pleural effusion), questionably actionable findings (such as atelectasis), or expected findings (increased perihilar markings). For patients who received multiple CXRs, the subsequent images were scored based on whether they were unchanged from the previous one, represented a new actionable finding, or if the finding was unrelated to bronchiolitis.

For the CBS calculation, variables collected 12 h after hospital admission included the highest respiratory rate (RR), highest heart rate (HR), highest temperature, lowest serum bicarbonate, lowest pH with the highest pCO_2_ ratio, and the worst Glasgow Coma Scale (GCS) score. For intubated patients, the score also incorporated the highest blood urea nitrogen (BUN) and lowest systolic blood pressure (SBP) values.

A list of collected variables is in Table A1 in the online supplement.

Medical documentation was complete for patients included in the study. Data was collected by physicians performing chart reviews of identified patients via EPIC, electronic medical record. Data was then input into Microsoft Excel prior to performing statistical analysis.

### 2.4. Statistical Analysis

A target sample size of 97 patients was determined a priori to achieve a power of 0.8, an alpha level of 0.05, and an effect size of a 2-day difference in LOS and LRS between the two groups. To evaluate differences in demographics and assess the association of antibiotic usage and CXRs between groups, a chi-square analysis or Fisher’s exact test was performed for categorical variables.

Given the non-parametric nature and non-normal distribution of the variables, the Mann-Whitney U test was used to analyze differences between CXR utilization and ICU-LOS and ICU-LRS. Additionally, the Mann-Whitney U test was applied to compare differences between actual and predicted values (actual minus predicted) for ICU-LOS and ICU-LRS between the two groups. This analysis was performed using GraphPad Prism version 10.3.0 (461) (GraphPad Software, LLC, 2024; www.graphpad.com, accessed on 17 February 2025).

## 3. Results

**Figure 1:**
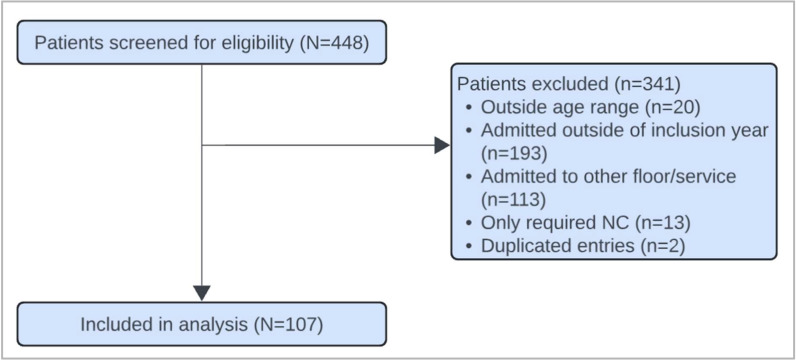
Consort diagram showing patient selection for analysis.

Of the 448 patients screened, 107 met inclusion criteria (Figure 1). The demographic characteristics of the cohorts are listed in Table 1. No significant differences were identified in key baseline demographics between the groups that might confound ICU-LOS and ICU-LRS. Of the 107 patients included in the study, 65 patients (61%) underwent CXR during their admission, while 42 patients (39%) did not receive a CXR. All three patients who were intubated received a CXR.

**Figure 2:**
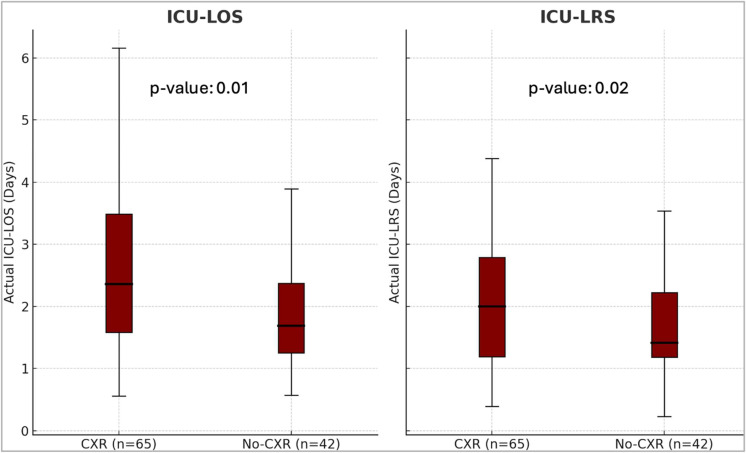
Box and whisker plots showing the difference in median observed ICU-LOS and ICU-LRS between the two groups. The median ICU-LOS and ICU-LRS were significantly shorter in the no-CXR group. Statistical analysis was performed using Mann-Whitney U testing. ICU = Intensive Care Unit, LOS = Length of stay, LRS = Level of Respiratory Support, CXR = Chest X-Ray.

Patients in the no-CXR group had a significantly shorter observed median ICU-LOS of 1.7 days (IQR: 1.3 to 2.4) compared to 2.4 days (IQR: 1.6 to 3.5) in the CXR group (*p* = 0.01). Similarly, the observed ICU-LRS was significantly shorter in the no-CXR group, 1.4 days (IQR: 1.2 to 2.2), compared to 2.0 days (IQR: 1.2 to 2.8) in the CXR group (*p* = 0.02) (Figure 2).

There was no significant difference in illness severity between the two groups. The predicted median ICU-LOS and ICU-LRS were 3.5 days (IQR: 2.7 to 4.2) and 2.8 days (IQR: 2.2 to 3.3), respectively, in the CXR group vs. 3.3 days (IQR: 2.5 to 4.1) and 2.6 days (IQR: 2.0 to 3.2) in the no-CXR group (*p* = 0.43 and *p* = 0.31).

**Figure 3:**
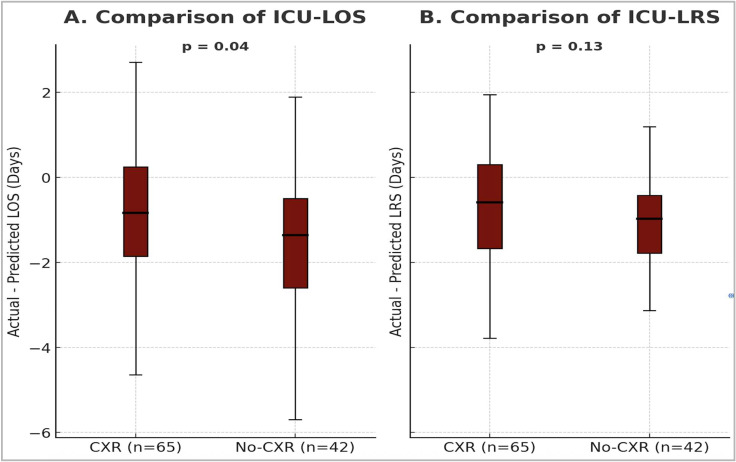
(**A**,**B**): Box and whisker plots showing differences in median ICU-LOS and ICU-LRS between the two groups. In (**A**), there was a significant difference in actual and predicted ICU-LOS between the groups. In (**B**), there was no significant difference in actual and predicted ICU-LRS between the two groups. ICU = Intensive Care Unit, LOS = Length of stay, LRS = Level of Respiratory Support, CXR = Chest X-Ray.

Actual ICU-LOS and ICU-LRS were significantly shorter than predicted values in both the CXR and no-CXR groups (Table 2). The median difference in ICU-LOS (actual minus predicted) was significantly shorter in the no-CXR group: –1.4 (IQR: –2.6 to –0.5) days, compared to –0.8 (IQR: –1.9 to 0.3) days in the CXR group (*p* = 0.04) (Figure 3). In contrast, the difference in median ICU-LRS between the two groups was not statistically significant: –0.6 (IQR: –1.7 to 0.3) days for the CXR group vs. –1.1 (IQR: –1.8 to –0.4) days for the no-CXR group (*p* = 0.13) (Figure 3).

Among those who received a CXR, 8 patients (12%) had actionable findings, 16 patients (25%) had possibly actionable findings, and 41 patients (63%) had expected findings. All patients with actionable findings received antibiotics (8/8), 44% (7/16) of those with questionable findings, and 24% (10/41) of those with expected bronchiolitis findings. Overall, antibiotic use was 38% (25/65) in the CXR group and 7% (3/42) in the no-CXR group. This difference was statistically significant (*p* = 0.0007), indicating an association with CXRs and higher antibiotic use (Table 3).

Of the 65 patients who received an initial CXR, 15 (23%) underwent repeat imaging. In most of these cases (87%), the repeat CXR confirmed the initial interpretation—whether the findings were significant, possibly significant, or non-significant. Only 2 patients (13%) were reclassified as having a significant finding on repeat imaging after an initially non-significant result.

Seven patients underwent echocardiography, 6 of whom also had CXRs. In the CXR group, 6 patients had echocardiograms, which revealed stable CHD or normal findings. In the no-CXR group, one patient underwent echocardiography, which revealed stable CHD. No patients in either group had a chest CT or lung ultrasound obtained.

## 4. Discussion

To our knowledge, this is the first study conducted in the ICU setting that evaluates the association of ordering routine CXRs on PICU outcomes in patients with critical bronchiolitis. Our findings demonstrate that obtaining a CXR on admission was associated with statistically significantly longer ICU-LOS and ICU-LRS, despite similar illness severity. This is consistent with previous studies which show the association of associated with longer hospital LOS with hospitalized patients who received CXRs [14] and confirms guidelines which recommend against routine CXRs for patients with bronchiolitis [1,2,3]. However, we believe the results of this study lend new hesitancy to the idea of performing routine CXRs for patients with severe bronchiolitis [1] and suggest that the overwhelming majority of those with critical bronchiolitis also do not benefit from CXRs.

On the minds of physicians performing CXRs is surely to confirm diagnosis, to look for complications, and to look for actionable items that can be managed in the ICU. More than 60% of our patients received a routine CXR, yet only 12% of those (8/65) had clearly actionable findings such as consolidation, effusion, or pneumothorax. An additional 25% had questionably actionable findings (atelectasis), which are common in viral illnesses and often misinterpreted as bacterial pneumonia. Consistent with prior literature, these nonspecific findings may have influenced increased antibiotic use [12,14]: 100% of patients with actionable CXRs received antibiotics, as did 43% with possibly actionable findings. Overall, antibiotic administration was five times higher in the CXR group than in those not imaged (38% vs. 7%). This difference was statistically significant, aligning with prior studies suggesting CXRs may drive unnecessary antibiotic exposure without improving outcomes [9,14,15,16,17].

Although the predicted ICU-LOS and ICU-LRS were similar between groups, the observed durations were significantly shorter in the no-CXR group. This difference warrants careful interpretation and may stem from several mechanisms. First, misinterpreting radiographic findings as a bacterial infection may delay the de-escalation of respiratory support or prolong pulmonary clearance treatments, despite serious bacterial infections being rare in bronchiolitis [1,8,18]. Second, providers may perceive radiographic abnormalities as indicators of more severe illness, prompting a more conservative approach and addition of antibiotics [12]. Third, CXR use may reflect physician bias toward diagnostic thoroughness, even in the absence of clinical deterioration [11,14]. This introduces the potential for reverse causality, wherein longer ICU stays and more complex courses prompted CXR use, rather than resulting from it, which can be difficult to ascertain based on the observational nature of this study. Alternatively, patients with prolonged ICU courses may represent a distinct subgroup—potentially with a different or overlapping diagnosis—that would not be fully captured by the CBS.

The relative differences between actual and predicted ICU-LOS remained greater in the no-CXR group, further reinforcing the observed association between CXR use and longer ICU stays. The median ICU-LOS and ICU-LRS in our cohort were significantly shorter than CBS predictions for both groups. This may be attributed to variations in institutional ICU-admission criteria, management strategies, clinical pathways, or regional differences in disease presentation. While supportive care is at the forefront of bronchiolitis management, there are nuanced differences in national guidelines regarding the use of pharmacologic therapy, oxygen thresholds, and respiratory support management that might affect the generalizability of this study’s findings [1,2,3,8]. One such example of these pathways present at our institution is an algorithm in which rapid weaning and subsequent discontinuation of HFNC support are commonly done on patients; a high-flow holiday [19,20]. This AAP initiative helps standardize the management at our institution and reduces practice variability at our center. This, however, may not be the practice at all institutions and may account for the differences in our median ICU-LOS and ICU-LRS when compared to the predicted numbers from the CBS.

Although CXRs revealed actionable findings in a minority of cases (12%), the low yield underscores the importance of more targeted imaging approaches. Prior studies have noted that hypoxia is a modest predictor of radiographic abnormalities [15,18], but widespread CXR use—even in non-intubated patients—remains common in PICUs [4]. Importantly, routine imaging not only contributes to cumulative radiation exposure in young children but also adds to healthcare costs without clear benefit in most cases [7]. These costs were not included in the study as they vary based on location, insurance, facility, and number of views, but remain an important factor in providing cost-effective healthcare. Future work should focus on identifying subpopulations (e.g., sudden unexplained deterioration, persistent hypoxemia, ongoing fever, or lack of clinical improvement) in whom CXRs may alter management [3,21].

Our findings reinforce the AAP’s recommendation against routine imaging in bronchiolitis, a recommendation intended for non-severe disease. The current guidelines from the AAP state that “Initial radiography should be reserved for cases in which respiratory effort is severe enough to warrant ICU admission or where signs of an airway complication (such as pneumothorax) are present [8]. The Canadian Paediatric Society echoes a similar sentiment in their position statement, in which chest radiography is considered “when the diagnosis of bronchiolitis is unclear, the rate of improvement is not as expected, or the severity of disease raises other diagnostic possibilities such as bacterial pneumonia” [18]. Our study specifically focused on the group that would qualify as severe disease to evaluate the relative burden of radiography and its impact on outcomes.

This study is limited by its single-center, retrospective design. Inherent limitations to this study design are the possibility of selection bias, unexplained confounders, and missing a subset of patients based on inadequate coding. We recognize that there may be institutional policies present as well as individual provider preferences that might affect our results, although at LLUCH, there is a Bronchiolitis Task Force that aims to reduce CXR use and follow AAP guidance for all levels of patients with bronchiolitis. The regional disease burden of our bronchiolitis population may limit the generalizability of these findings, as developed countries have lower bronchiolitis mortality rates than developing countries [22]. While the CBS standardizes illness severity, it may not entirely capture nuances of clinical decision-making or identify subtle clinical signs, but it remains a good starting point. The CBS also does not include some known variables associated with illness severity (prematurity, work of breathing, multiple viruses, etc.). Still, it is a more appropriate way to adjust for disease severity in low-mortality illnesses, such as bronchiolitis. Due to the low-morbidity nature of bronchiolitis and retrospective study design, long-term outcomes were not evaluated in this study. We also did not assess the timing of management strategies following CXR findings, such as the initiation of antibiotics, additional respiratory therapies, or escalation of respiratory support. These aspects warrant investigation in future prospective studies.

## 5. Conclusions

Obtaining CXRs in critically ill bronchiolitis patients may be associated with longer ICU length of stay, ICU length of respiratory support, and increased use of antibiotics despite similar illness severity. Future research should focus on identifying criteria for targeted imaging in subsets of bronchiolitis patients.

## Figures and Tables

**Table 1:** Demographic Characteristics of the CXR and no-CXR group. There was no significant difference between the demographics of the two groups. Statistical testing was performed with Chi-Square Analysis ^1^. N = Total number of patients, F = Female; M = Male; AA = African American; PMHX = Past Medical History; BPD = Bronchopulmonary dysplasia; CHD = Congenital Heart Disease; CXR = Chest X-Ray.

Demographics	Group	CXR	No CXR	*p*-Value ^1^
N		65	42	
Gender	F	26 (40%)	17 (40%)	0.96
	M	39 (60%)	25 (60%)	
Age on Admission		12.1 months	12.0 months	N/A
Ethnicity	Asian	4 (6%)	2 (5%)	0.61
	AA	6 (14%)	2 (5%)	
	Caucasian	15 (23%)	15 (36%)	
	Hispanic	40 (62%)	22 (52%)	
	Other	1 (2%)	1 (2%)	
PMHx	Prematurity	21 (32%)	10 (24%)	0.51
	BPD	1 (2%)	1 (2%)	1
	Atopy	9 (14%)	10 (24%)	0.3
	CHD	8 (12%)	4 (10%)	0.9
Day of Illness	<3 days	38 (58%)	24 (57%)	1
	>3 days	27 (42%)	18 (43%)	
Location of Admission	PICU	39 (60%)	29 (69%)	0.9
	SDICU	16 (25%)	7 (17%)	0.8
	CTICU	10 (15%)	6 (14%)	1

**Table 2:** Data are presented as median days (25–75% IQR) unless otherwise indicated. The CXR group had significantly longer ICU-LOS and ICU-LRS than the no-CXR group despite no difference in predicted ICU-LRS and ICU-LOS. The ICU-LOS and ICU-LRS for both groups were significantly shorter than predicted by the critical bronchiolitis score. Statistical analysis performed utilizing the Mann-Whitney U test ^1^. ICU = Intensive Care Unit, LRS = Level of Respiratory Support, CXR = Chest X-Ray.

	LOS			LRS		
	CXR Y	CXR N	*p*-value ^1^	CXR Y	CXR N	*p*-value ^1^
Actual	2.3 (1.6–3.5)	1.7 (1.2–2.4)	0.01	2.0 (1.2–2.8)	1.4 (1.2–2.2)	0.018
Predicted	3.5 (2.7–4.2)	3.3 (2.5–4.1)	0.43	2.8 (2.2–3.3)	2.6 (2.0–3.2)	0.31

**Table 3:** Observed use of antibiotics in patients with CXR and No-CXR and the association of CXR findings with % antibiotic usage. Statistical analysis performed using independent two-sample *t*-test *, *p* = 0.0007.

	n (%)	Antibiotic (n, %)	No Antibiotic (n, %)
CXR	65 (60.7%)	25 (38.5%) *	40 (61.5%)
CXR—Actionable	8 (7.5%)	8 (100%)	0 (0%)
CXR—Questionable	16 (15%)	7 (43.8%)	C9 (56.3%)
CXR—Expected	41 (38.3%)	10 (24.4%)	31 (75.6%)
No CXR	42 (39.3%)	3 (7.1%) *	39 (92.9%)
Total (N)	107 (100%)	28 (26.2%)	79 (73.8%)

## Data Availability

De-identified data underlying the findings of this study are available from the corresponding author upon reasonable request. The data will be provided in an Excel format, subject to institutional and ethical guidelines.

## Abbreviations

The following abbreviations are used in this manuscript:

PICU	Pediatric intensive care unit
SDICU	Step-down intensive care unit
PCTICU	Pediatric cardiac intensive care unit
ICU	Intensive care unit
LOS	Length of stay
CBS	Critical Bronchiolitis Score
LRS	Level of respiratory support
HR	Heart rate
RR	Respiratory rate
GCS	Glasgow Coma Scale
pCO_2_	Partial pressure of carbon dioxide
SBP	Systolic blood pressure
BUN	Blood urea nitrogen
HFNC	High-flow nasal cannula
NIMV	Non-invasive mechanical ventilation
IMV	Invasive mechanical ventilation
CXR	Chest X-ray
IQR	Interquartile range
AAP	American Academy of Pediatrics

## Appendix A

**Table A1:** List of collected data variables.

Variable	Units	Concept
Demographic data	-	Patient characteristics
Past medical history	-	Medical conditions prior to admission
Past surgical history	-	Surgical procedures prior to admission
Past family history of atopy in a first-degree relative	-	Family predisposition to atopic conditions
Home-oxygen use	Yes/No	Pre-admission oxygen therapy at home
Day-of-illness on presentation	Days	Duration of illness before presentation
Month and year of presentation	Month/Year	Temporal factors of admission
Presence of viral infection	Names of virus identified	Confirmed viral etiology
ICU-LOS	Days	Length of ICU stay
Hospital LOS	Days	Length of hospital stay
HFNC use	Yes/No, L/Kg, Days	Duration of respiratory support
NIMV use (including nCPAP)	Yes/No, Highest PIP/PEEP/RR/FiO2, Days	Duration of respiratory support
IMV use (including HFOV)	Yes/No, Highest PIP/PEEP/MAP/RR/FiO2, Days	Duration of respiratory support
CXR use	Yes/No	Diagnostic modality
Other imaging- US, Echocardiography, CT	Yes/No	Diagnostics
Antibiotic use	Yes/No	Clinical management
Nebulizations—Albuterol, 3%, racemic epinephrine	Yes/No	Adjunctive therapies
Highest RR	Breaths per minute	Vital signs
Highest HR	Beats per minute	Vital signs
Highest temperature	°C or °F	Vital signs
Worst GCS	GCS score	Clinical decision tool
Lowest serum bicarbonate	mmol/L	Measure of dehydration
Lowest pH/highest pCO_2_ ratio	pH, mmHg	Measure of respiratory acidosis
Highest BUN	mg/dL	Measure of dehydration
Lowest SBP	mmHg	Vital signs
